# Tuberculose multifocale chez les immunocompétents

**DOI:** 10.11604/pamj.2016.24.13.6030

**Published:** 2016-05-04

**Authors:** Amel Rezgui, Fatma Ben Fredj, Anis Mzabi, Monia Karmani, Chadia Laouani

**Affiliations:** 1 Service de Médecine Interne, CHU Sahloul, Sousse, Tunisie

**Keywords:** Tuberculose multifocale, immunocompétent, atteinte pulmonaire, Multifocal tuberculosis, immunocompetent, lung damage

## Abstract

La tuberculose multifocale est définie par la l'atteinte d'au moins deux sites extra-pulmonaires associée ou non à une atteinte pulmonaire. On se propose d’étudier les différentes caractéristiques cliniques et évolutives de la tuberculose multifocale à travers une étude rétrospective de 10 cas. Parmi 41 cas de tuberculose colligés entre 1999 et 2013. Dix patients avaient une tuberculose multifocale, soit 24% des patients. Il s'agissait de 9 femmes et 1 homme d’âge moyen à 50 ans (30-68 ans). Nos patients étaient tous correctement vaccinés par le BCG. Un bilan à la recherche d'une éventuelle immunodépression fait pour tous les patients était négatif. Il s'agissait d'une tuberculose ganglionnaire dans 7 cas, digestive dans 3 cas, péricardique dans 2 cas, ostéo-articulaire dans 2 cas, cérébrale dans 1 cas, urinaire dans 2 cas, uro-génitale dans 4 cas, surrénalienne dans 1 cas, cutanée dans 1 cas et musculaire dans 1 cas. Tous nos patients ont bénéficié d'un traitement antituberculeux pour une durée moyenne de 10 mois avec bonne évolution. La tuberculose multifocale est une des maladies à diagnostic difficile. Elle peut toucher les immunocompétents mais son pronostic est souvent bon. Un traitement anti-tuberculeux doit être instauré le plus rapidement possible pour éviter les séquelles.

## Abstract (en)

Multifocal tuberculosis is defined as the presence of lesions affecting at least two extrapulmonary sites, with or without pulmonary involvement. This retrospective study of 10 cases aims to investigate the clinical and evolutionary characteristics of multifocal tuberculosis. It included 41 cases with tuberculosis collected between 1999 and 2013. Ten patients had multifocal tuberculosis (24%): 9 women and 1 man, the average age was 50 years (30-68 years). Our patients were correctly BCG vaccinated. The evaluation of immunodepression was negative in all patients. 7 cases had lymph node tuberculosis, 3 cases digestive tuberculosis, 2 cases pericardial tuberculosis, 2 cases osteoarticular tuberculosis, 1 case brain tuberculosis, 2 cases urinary tuberculosis, 4 cases urogenital tuberculosis, 1 case adrenal tuberculosis, 1 case cutaneous and 1 case muscle tuberculosis. All patients received anti-tuberculosis treatment for a mean duration of 10 months, with good evolution. Multifocal tuberculosis is difficult to diagnose. It can affect immunocompetent patients but often has good prognosis. Anti-tuberculosis therapy must be initiated as soon as possible to avoid sequelae.

## Introduction

En raison de sa forte incidence, la tuberculose (TBC) constitue un problème majeur de la santé publique dans les pays en développement notamment en Tunisie. La tuberculose multifocale est définie par l'atteinte d'au moins deux sites extra-pulmonaires associée ou non à une atteinte pulmonaire. Les formes multifocales sont rares et représentent 9 à10% des localisations extra-pulmonaires. Leur pronostic est mauvais avec un taux de mortalité de 16 à 25% selon les auteurs [1–3]. La tuberculose multifocale survient habituellement chez les immunodéprimés, mais elle peut toucher les immunocompétents. A ce propos nous rapportons 10 cas de TBC multifocale avec une revue de la littérature.

## Méthodes

Il s'agit d'une étude descriptive rétrospective des dossiers de patients immunocompétents diagnostiqués porteurs d'une tuberculose entre 1999 et 2013 dans le service de médecine interne au CHU Sahloul de Sousse, Tunisie. Le diagnostic a été retenu devant une confirmation histologique et/ou bactériologique ou devant la convergence d'un faisceau d'arguments épidémio-cliniques et /ou radiologiques et/ou anatomopathologiques suggestifs avec une preuve thérapeutique. Les données cliniques, paracliniques, thérapeutiques et évolutifs ont été recueillis sur une fiche de renseignement préétablie. L'analyse de ces paramètres a été faite grâce à un logiciel de statistiques médicales SPSS.

## Résultats

Quarante et un cas de tuberculose ont été colligés. Dix patients avaient une tuberculose multifocale, soit 24% des patients. Il s'agissait de 9 femmes et 1 homme d’âge moyen à 50 ans (30- 68 ans). Nos patients étaient tous correctement vaccinés par le BCG et n‘étaient sous aucun traitement immunosuppresseur. Un bilan à la recherche d'une éventuelle immunodépression fait pour tous les patients était négatif, à savoir un dosage pondéral des immunoglobulines, un comptage des lymphocytes, une électrophorèse des protides et une sérologie HIV. Tous les patients avaient un bon état nutritionnel. Deux patientes étaient diabétiques. Aucun patient n'avait la notion d'un contage tuberculeux. Un seul patient avait une localisation pulmonaire. Il s'agissait d'une localisation double pour 8 patients et triple pour 2 patients. Il s'agissait d'une tuberculose ganglionnaire dans 7 cas, digestive dans 3 cas, péricardique dans 2 cas, ostéo-articulaire dans 2 cas, cérébrale dans 1 cas, urinaire dans 2 cas, uro-génitale dans 4 cas, surrénalienne 1 cas, cutanée dans 1 cas et musculaire dans 1 cas.

Le diagnostic de la tuberculose a été apporté par un examen histologique dans 9 cas montrant un granulome épithéloïde et giganto-cellulaire avec une nécrose caséeuse, et/ou l'isolement de bacilles de Kock dans 2 cas (Tableau 1). Le délai diagnostic a varié entre 1 et 6 mois avec une moyenne à 4 mois.

**Tableau 1:** Circonstances de découverte, localisations et moyens diagnostiques de la tuberculose multifocale

Cas	Circonstances de découverte	Localisation	Moyens de confirmation diagnostique
1	Fièvre, retention urinaire, douleurs abdominales	Urogénitale et digestive (iléocoecale et pancréatique)	RBK et culture des urines positives, biopsie colique: granulome gigantocellulaire et épithéloïde avec nécrose caséeuse (GGCENC)
2	Fièvre et hypersudation nocturne, douleurs abdominales	Urogénitale et digestive	Biopsies ovarienne, mésentérique et péritonéale: GGCENC
3	Hypersudation nocturne, adénopathies cervicales, lombalgies	Ganglionnaire, ostéoarticulaire et péricardique	Biopsie ganglionnaire: GGCENC
4	Hypersudation, adénopathies cervicales	Ganglionnaire et surrenalienne	biopsie ganglionnaire: GGCENC
5	Fièvre et hypersudatioon nocturne	Ganglionnaire et cérébrale	IRMc: lésions à centre nécrotique, biopsie ganglionnaire: GGCENC
6	Douleurs de la hanche gauche, collection de la cuisse droite	Sacroiliaque gauche et quadriceps droit	Biopsie musculaire: GGCENC
7	Asthénie et fièvre	Ganglionnaire et rénale	Biopsie ganglionnaire et pièce de nephrectomie: GGCENC
8	Fièvre, adénopathies profondes	Cutanée, pulmonaire et ganglionnaire profondes	Epreuve thérapeutique
9	Fièvre, polyadénopathie profonde et périphérique	Ganglionnaire et digestive	RBK et culture du liquide d'ascite positives
10	Adénopathies cervicales	Ganglionnaire et péricardique	Biopsie ganglionnaire: GGCENC

Tous nos patients ont bénéficié d'un traitement antituberculeux pour une durée moyenne de 10 mois (8 mois-18 mois). L’évolution était favorable sur le plan clinique, biologique et radiologique pour 9 patients. Une seule patiente avait rechuté avec apparition d'une troisième localisation due à une mal observance thérapeutique. Elle a bien évolué après prolongation de la durée de l'antibiothérapie à 18 mois. Le taux d'iatrogénie était à 40% (une hyper uricémie: 3 cas et/ou une cytolyse hépatique modérée: 2 cas).

## Discussion

La tuberculose diffuse ou multifocale représente 10% des atteintes extra pulmonaires. Elle est responsable d'un tiers de la mortalité chez les malades porteurs du VIH. Elle peut toucher aussi les immunocompétents. Les formes multifocales sont rares et représentent 9 à10% des localisations extra-pulmonaires. Leur pronostic est mauvais avec un taux de mortalité de 16 à 25% selon les auteurs [1–3]. On en trouve quelques cas isolés dans la littérature [4]. Les plus grandes séries publiées sont celle de Denis et celle de Ghorbel comportant les deux 47 cas [1, 5]. Cela suggère la rareté de cette forme dans les pays industrialisés. Les formes multifocales sont plutôt étudiées de manière globale dans les séries intégrant toutes les formes cliniques [6]. Le contage tuberculeux est le plus souvent retrouvé dans 20 à 60% des cas selon les séries [7]. Dans notre série, aucun cas de contage tuberculeux n’ a été noté.

Un délai long pour le recours à l'hôpital, donc au diagnostic, est souvent rapporté dans les études africaines. C’était le cas dans notre étude (en moyenne, 4 mois) et cela peut en partie expliquer la diffusion des lésions. Plusieurs facteurs peuvent expliquer ce retard au diagnostic: erreur diagnostique de la part du personnel soignant, problèmes économiques et culturels, dont une sous-information de l'entourage [8].

Il a été clairement démontré que le risque de développer des atteintes extra pulmonaires est proportionnel au degré du déficit immunitaire [9]. Dans notre étude, la localisation était double ou triple en dehors de tout déficit immunitaire. Plusieurs hypothèses ont été avancées pour expliquer la survenue de cette forme grave de tuberculose chez les immunocompétents. Certains auteurs ont pu établir une relation entre la tuberculose diffuse et l'intensité de la transmission dans la collectivité [10]. D'autres impliquent la malnutrition comme facteur favorisant [8]. Cathérinot [11] a décrit le syndrome de la susceptibilité mendélienne aux infections mycobactériennes par l'existence de défauts de l'axe interleukine 12-interféron gamma et exposant à la tuberculose diffuse.

La localisation ganglionnaire était la plus fréquente dans notre série, ainsi que dans les différentes séries de la littérature, contribuant facilement au diagnostic par son accessibilité a l'examen anatomo-pathologique. Il est à noter la sévérité de la localisation cérébro-méningée du fait de la difficulté diagnostique en dehors d'une autre localisation tuberculeuse associée et du taux élevé de mortalité variant entre 30 et 50%. Dans notre étude, le patient diagnostiqué porteur d'une tuberculose méningée avait en plus des adénopathies cervicales dont la biopsie a permis de poser le diagnostic.

L'atteinte ostéoarticulaire est noté dans 1 à 5% des cas selon les séries. C'est une localisation souvent sous-diagnostiqué surtout si elle inaugure le tableau expliquant parfois le retard diagnostique. Il s'agit souvent d'une spondylodiscite, plus rarement d'une arthrite périphérique [12]. L'atteinte cutanée a intéressé un seul patient dans notre série, elle est classée comme étant la cinquième localisation selon la littérature et représente une forme à diagnostic facile [13, 14].

L’évolution sous traitement bien conduit, démarré le plus précocement possible, est souvent favorable permettant d’éviter les séquelles. Une de nos patients a rechuté avec apparition d'une autre localisation tuberculeuse due uniquement à une mal observance thérapeutique. Il faut donc éduquer les patients sur la nécessité d'une observance thérapeutique et penser, en cas de rechute, aux problèmes de résistances du bacille de Kock aux anti-tuberculeux.

## Conclusion

La tuberculose multifocale est une forme grave touchant habituellement les immunodéprimés ayant déjà une localisation pulmonaire. Cependant, elle peut toucher des sujets immunocompétents en dehors de toute localisation pulmonaire. Il est donc nécessaire de faire systématiquement un bilan exhaustif de dissémination du germe de la tuberculose et ce pour une meilleure prise en charge. Le pronostic est souvent favorable, dépendant du type de l'atteinte et de la précocité de l'instauration des anti-tuberculeux.

### Etat des connaissances actuelles sur le sujet

- La tuberculose (TBC) constitue un problème majeur de la santé publique dans les pays en développement notamment en Tunisie;
- La tuberculose multifocale est définie par l'atteinte d'au moins deux sites extra-pulmonaires associée ou non à une atteinte pulmonaire;
- Les formes multifocales sont rares et représentent 9 à10% des localisations extra-pulmonaires.

### Contribution de notre étude à la connaissance

- La tuberculose multifocale est une forme de tuberculose pouvant se voir chez les immunodéprimés. Peu d’études se sont intéressées à cette forme chez les immunocompétents comme le fait notre étude;
- Il est intéressant de voir le profil épidémiologique, clinique et évolutif des patients immunocompétents développant une tuberculose multifocal, afin d'améliorer leur prise en charge;
- On rappelle par cette étude que les immunocompétents ne sont pas à l'abri des formes graves et diffuses de tuberculose, ce qui incite les cliniciens à faire le bilan lésionnel devant une tuberculose à la recherche d'autres localisations même s'il ne s'agit pas d'un immunodéprimé. Et ce à cause d'une probable intensité élevée de transmission du BK dans les pays en voie de développement.
